# The Utility of Oncotype DX for Adjuvant Chemotherapy Treatment Decisions in Estrogen Receptor-positive, Human Epidermal Growth Factor Receptor 2-negative, Node-negative Breast Cancer

**DOI:** 10.7759/cureus.7269

**Published:** 2020-03-14

**Authors:** Hirah Rizki, Christopher Hillyar, Omar Abbassi, Sascha Miles-Dua

**Affiliations:** 1 Breast Surgery, Mid Essex Hospitals National Health Service (NHS) Trust, Broomfield, GBR; 2 Surgery, Barts Health National Health Service (NHS) Trust, London, GBR; 3 Surgery, Mid Essex Hospitals National Health Service (NHS) Trust, Broomfield, GBR

**Keywords:** oncotype dx, breast cancer, predict, npi, recurrence risk

## Abstract

Introduction

Breast cancer is the most common cancer diagnosis in the UK. Recently, there has been a reduction in breast cancer-specific mortality and recurrence attributed, in part, to the delivery of adjuvant chemotherapy. The National Institute for Health and Care Excellence (NICE) recommends the use of genetic profiling with Oncotype DX (ODX) to guide decisions to offer adjuvant chemotherapy after surgery in intermediate-risk early breast cancer patients. This study aimed to evaluate the utility of ODX testing in routine clinical practice in a National Health Service (NHS) hospital.

Methods

Consecutive early breast cancer patients, identified through the multidisciplinary team (MDT) records, treated in our institution over 12 months (October 2017-September 2018) were included. PREDICT and Nottingham prognostic index (NPI) scores (from online clinicopathological recurrence risk tools) were calculated for each patient, and ODX data obtained through Genomic Health, Inc. (Redwood City, California). Patients were divided into two groups, those that underwent ODX testing (ODX group) and those that did not (non-ODX group). Descriptive statistics were used to analyse patient and tumour characteristics. The Gaussian distribution of each data set was assessed using the Anderson-Darling test. For comparisons between patient groups, the non-parametric equivalent of the two-tailed t-test (Mann-Whitney) was used. Dichotomous variables (e.g. chemotherapy decisions) were compared using chi-squared tests.

Results

One-hundred thirty-three patients (mean age 62 years) treated for 152 early breast cancers, were included in the final analysis. Breast cancers in the ODX group were of greater median tumour size (24 vs 16 mm; P<0.0001) and higher median tumour grade (3 vs 2; P<0.0001). PREDICT scores (3 vs 1, P<0.0001) and NPI scores (3.40 vs 2.30, P<0.0001) for the ODX group were also significantly higher than the non-ODX group. A greater proportion of patients were offered chemotherapy in the ODX group (39.9% vs 6.9%, P<0.001). However, for the PREDICT-calculated intermediate-risk patients, ODX testing resulted in a lower proportion of patients being offered chemotherapy compared to the intermediate-risk patients who were not genetically profiled (54.5% vs 83.3%, P=0.3547), although this result was not statistically significant.

Conclusions

Patients selected for ODX testing were younger, with significantly higher-grade and larger-sized tumours compared to patients not selected for genetic profiling. ODX testing significantly impacted the delivery of chemotherapy, as the recurrence score generated through ODX testing guided the final decision.

## Introduction

Breast cancer is the most common cancer diagnosis in the UK, with approximately 55,000 new cases diagnosed each year [1]. The specific mortality and recurrence rates for breast cancer have improved over recent years, due, in part, to the use of adjuvant chemotherapy treatment. Indeed, a meta-analysis from the Early Breast Cancer Trialists' Collaborative Group that assessed long-term outcomes in 100,000 women reported that the use of adjuvant chemotherapy conferred a significant reduction in breast cancer mortality and recurrence rates [2]. However, the use of chemotherapy needs to be carefully considered on an individual basis because these drugs are associated with toxic side effects, such as myelosuppression, neutropenic sepsis, cardiac toxicity, and neuropathy [3].

To assist with the decision to treat a patient with adjuvant chemotherapy, scoring systems have been developed which estimate a patient's composite risk of recurrence and mortality. These scoring systems identify patients with a higher risk of recurrence for whom the benefits of chemotherapy are most likely to outweigh the potential risks and side effects. PREDICT and the Nottingham prognostic index (NPI) are two such scoring systems [4,5]. The risk estimates they produce are based on clinicopathological factors, including age at diagnosis, lymph node status, tumour size, tumour grade, and hormone receptor status.

However, breast cancer is a heterogeneous, phenotypically diverse disease with varied behaviours and responses to chemotherapy. PREDICT and NPI provide little information regarding how individual cancers will respond to chemotherapy. In contrast, genetic profiling tests, such as Oncotype DX (ODX), assess the activity of specific genes that play an important role in the response of a particular cancer to chemotherapy [6].

ODX is a 21-gene assay that generates a recurrence score (RS) that predicts the 10-year risk of breast cancer recurrence and response to chemotherapy treatment. The Trial Assigning Individualized Options for Treatment (TAILORx) trial demonstrated that in individuals with a high RS, the administration of adjuvant chemotherapy significantly reduces cancer recurrence and mortality, while patients with a low RS received no additional benefit from chemotherapeutic treatment [7]. The National Institute for Health and Care Excellence (NICE) recommends the use of ODX to guide decision-making with regards to offering adjuvant chemotherapy after surgery [8]. NICE advocates the utilisation of ODX testing in estrogen receptor (ER)-positive, human epidermal growth factor receptor 2 (HER2)-negative, node-negative early breast cancer only if patients 'have an intermediate-risk of distant recurrence using a validated tool such as PREDICT or the NPI and the information provided by the ODX testing will inform adjuvant chemotherapy treatment decisions.

This study aimed to evaluate the utility of ODX testing for ER-positive, HER2-negative, node-negative early breast cancer in routine clinical practice in the National Health Services (NHS). To achieve this, our primary objective was to evaluate differences in the clinicopathological features of a cohort of patients who underwent ODX testing compared with a cohort of patients who were not selected for genetic profiling. The secondary objective was then to assess the impact of ODX testing on adjuvant chemotherapy treatment decisions by comparing differences in the delivery of chemotherapy between patients selected for ODX testing and those that were not selected for ODX testing.

## Materials and methods

Patient population

This study included consecutive early breast cancer patients treated in our institution over 12 months from 1st October 2017 to 30th September 2018. Inclusion criteria: 1) patients with early breast cancer who underwent primary surgery with curative intent; 2) ER-positive and HER2-negative breast cancers; 3) node-negative disease on sentinel lymph node biopsy (SLNB), and 4) patients with final histology results discussed at the local multidisciplinary team (MDT) meeting. Exclusion criteria: 1) patients with an in-breast recurrence; 2) patients that did not undergo an SLNB; and 3) patients with ductal carcinoma in situ (DCIS) only.

Data collection

Patients were identified through MDT records over the study period. Data were collected on patient demographics and clinicopathological features from electronic patient notes and histology reports. PREDICT, and NPI scores were calculated for each patient using online tools [9,10]. ODX data was obtained through Genomic Health, Inc. (Redwood City, California) and MDT records.

Patient Cohorts

All patients who met the inclusion criteria were divided into two groups. The first group included patients that underwent ODX testing (ODX group) and the second group included patients that did not have ODX testing or any other genetic profiling performed on their cancers (non-ODX group).

Data analysis

Descriptive statistics were used to analyse patient and tumour characteristics. The Gaussian distribution of each data set was assessed using the Anderson-Darling test. For comparisons between patient groups, the non-parametric equivalent of the two-tailed t-test (Mann-Whitney) was used. Dichotomous variables (e.g. chemotherapy decisions) were compared using non-parametric chi-squared tests. Statistical tests were performed using GraphPad Prism 8.1.

Patients were risk-stratified into high, intermediate and low-risk groups based on their score for chemotherapy benefit, prognosis and risk of recurrence as generated by PREDICT, NPI, and the Recurrence Score (RS) from ODX testing, respectively. The thresholds for risk stratification by RS, PREDICT, and NPI were based on our institutions' current practice, which is evidence-based [4,7,11].

## Results

Patient characteristics

This study included 134 consecutive patients; however, one patient was excluded due to incomplete data, resulting in a total of 133 patients (with a mean age of 62 years) being included in the final analysis. These patients were treated for 152 ER-positive, HER2-negative, node-negative early breast cancers. Of the 133 patients, the proportion of patients presenting symptomatically compared to those with breast cancer detected through national screening was not significantly different (51.1% and 48.9%, respectively). One-hundred nineteen out of 133 patients had a single tumour (89.5%) and 14/133 patients had multifocal disease (10.5%). The median tumour size was 20 mm and the median tumour grade was 2. Patient and tumour characteristics are summarised in Table 1.

**Table 1:** Patient and tumour characteristics ODX group, patients with cancers that were genetically profiled through Oncotype DX testing; non-ODX group, patients that did not have Oncotype DX testing. Abbreviation: Oncotype DX (ODX)

	All patients	ODX group	Non-ODX group
Patients, N	133	46	87
Age (years), mean	62	56	65
Disease identified through symptoms, n (%)	68 (51.1%)	32 (69.6%)	36 (41.4%)
Disease detected through screening, n (%)	65 (48.9%)	14 (30.4)	51 (58.6%)
Single-site disease, n (%)	119 (89.5%)	38 (82.6%)	81 (93.1%)
Multifocal disease, n (%)	14 (10.5%)	8 (17.4%)	6 (6.9%)
Tumours, n	152	57	95
Tumour size (cm), median	2.0	2.4	1.6
T1a (<5mm), n (%)	3 (1.9%)	1 (1.8%)	2 (2.1%)
T1b (<10mm), n (%)	16 (10.2%)	3 (5.3%)	13 (13.7%)
T1c (<20mm), n (%)	52 (33.1%)	13 (22.8%)	39 (41.1%)
T2 (>20mm), n (%)	72 (45.9%)	32 (56.1%)	40 (42.1%)
T3 (>50mm), n (%)	9 (5.9%)	8 (14.0%)	1 (1.1%)
T4 (>50mm with chest wall or skin invasion), n (%)	0 (0.0%)	0 (0.0%)	0 (0.0%)
Tumour grade, median	2	3	2
Grade 1, n (%)	28 (18.4%)	2 (3.5%)	26 (27.4%)
Grade 2, n (%)	81 (53.3%)	26 (45.6%)	55 (57.9%)
Grade 3, n (%)	43 (28.3%)	29 (50.9%)	14 (14.7%)

Characteristics of patients selected for Oncotype DX testing

**Figure 1:**
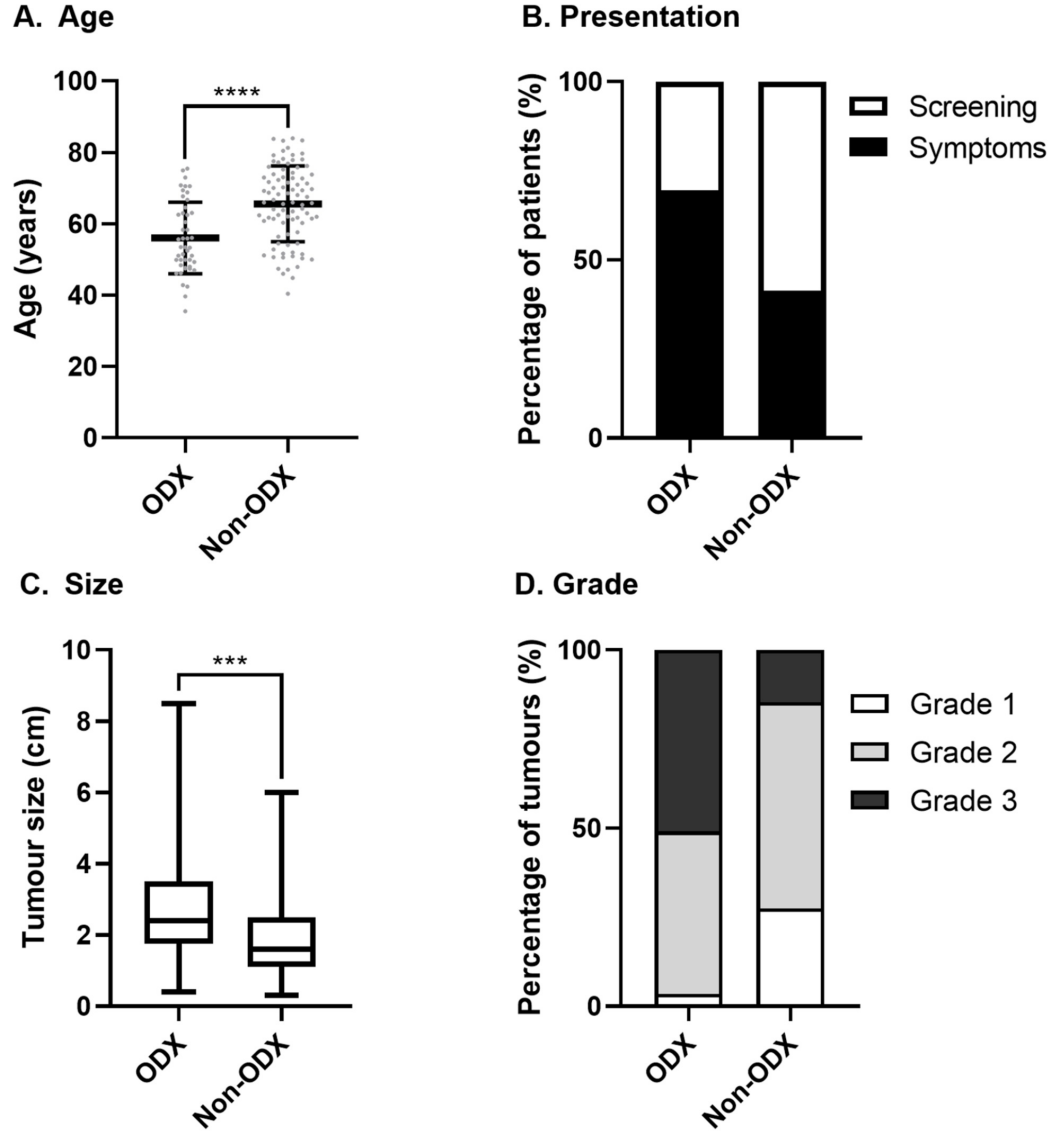
Patient and tumour characteristics of patients selected for Oncotype DX testing compared to patients not genetically profiled A) Mean age of patients in the ODX and non-ODX groups (Mann-Whitney, P<0.0001); B) percentage of patients in the ODX and non-ODX groups with symptomatic presentation or identification of cancer through national screening (chi-squared, P<0.0001); C) median tumour size (cm) of tumours for patients in the ODX and non-ODX groups (Mann-Whitney, P<0.001); and D) percentage of tumours classified as Grade 1, 2, or 3 for patients in the ODX and non-ODX groups (chi-squared, P<0.0001); ODX group, patients with cancers that were genetically profiled through Oncotype DX testing; non-ODX group, patients that did not have Oncotype DX testing.

Of the 133 patients, 46 patients were selected to undergo genetic profiling through ODX testing of their cancers (ODX group). The remaining 87 patients did not undergo genetic profiling and constituted the non-ODX group. Table 1 summarises the patient characteristics of the ODX and non-ODX groups. Patients in the ODX group were younger (mean age: 56 vs 65 years; P<0.0001) and were more likely to receive a cancer diagnosis due to the presentation of symptoms rather than by detection through breast cancer screening in comparison to patients in the non-ODX group (69.6% vs 41.4%; P<0.0001). No statistically significant differences in terms of single-site disease and multifocal disease were observed between the two groups. In the ODX group, the characteristics of the tumours were more severe, including greater median tumour size (24 mm vs 16 mm; P<0.0001) and higher median tumour grade (3 vs 2; P<0.0001) in comparison to tumours that were not genetically profiled (non-ODX group). Figure 1 illustrates the differences in age, presentation, size and grade of tumours for patients in the ODX and non-ODX groups.

**Figure 2:**
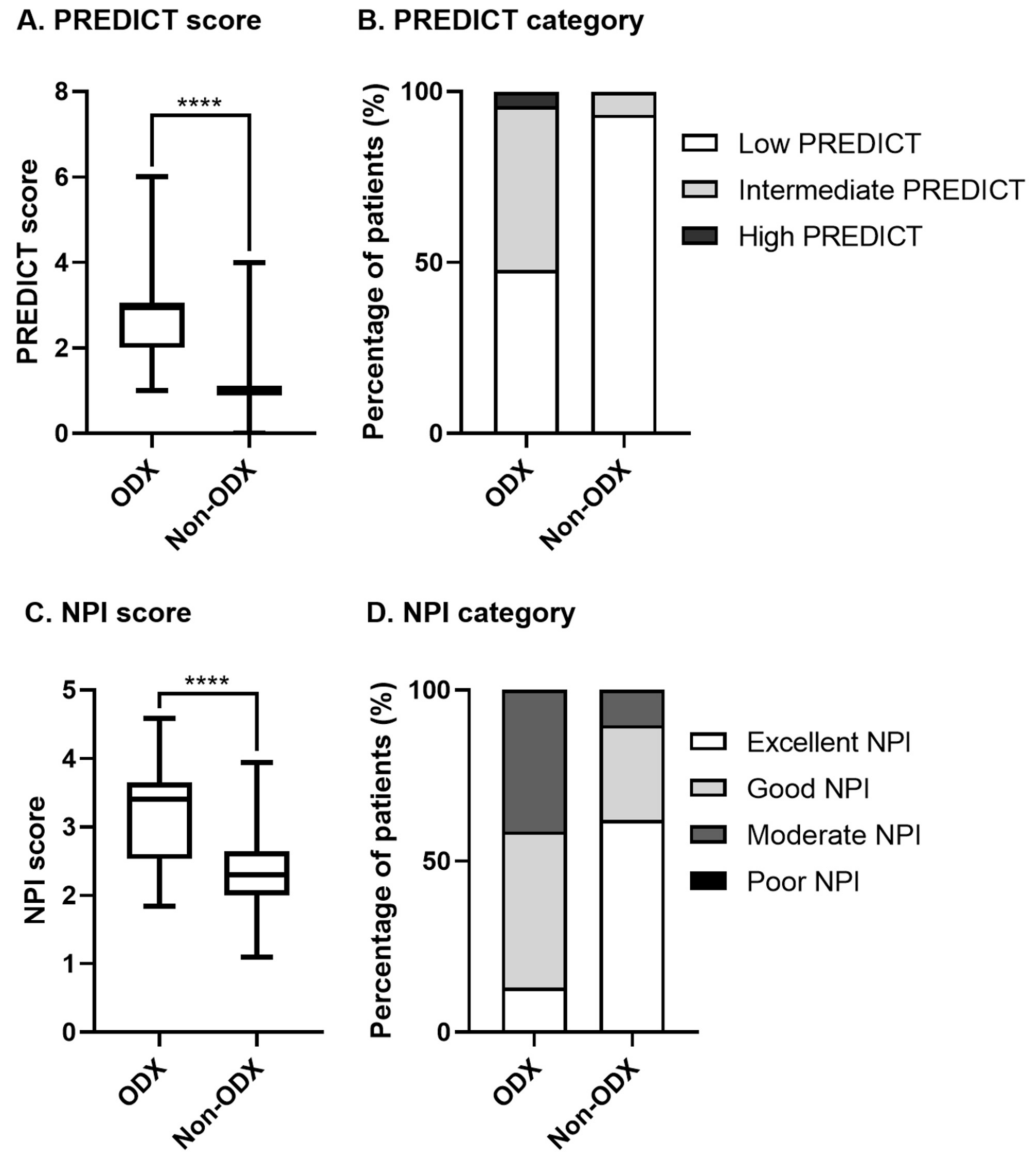
PREDICT and Nottingham prognostic index (NPI) scores for patients selected for Oncotype DX (ODX) testing compared to patients not genetically profiled A) Median PREDICT score for patients in the ODX and non-ODX groups (Mann-Whitney, P<0.0001); B) proportion of low, intermediate, or high PREDICT scores for patients in the ODX and non-ODX groups (chi-squared, P<0.0001); C) median NPI score for patients in the ODX and non-ODX groups (Mann-Whitney, P<0.0001 ); and D) proportion of excellent, good, moderate, or poor NPI scores for patients in the ODX and non-ODX groups (chi-squared, P<0.0001); ODX group, patients with cancers that were genetically profiled through Oncotype DX testing; non-ODX group, patients that did not have Oncotype DX testing; for patients with multifocal disease, the highest PREDICT or NPI score was used.

Statistically significant differences were also observed for PREDICT and NPI scores between the ODX and non-ODX groups. PREDICT scores (3 vs 1, P<0.0001) and NPI scores (3.40 vs 2.30, P<0.0001) were significantly higher for the ODX group in comparison to the non-ODX group. 52.1% of patients in the ODX group had intermediate to high PREDICT scores, while only 6.8% of patients in the non-ODX group fell into this intermediate PREDICT risk category (P<0.0001). 41.3% of patients in the ODX group had moderate NPI risk, while only 10.3% of patients in the non-ODX group had similar NPI scores (P<0.0001). Figure 2 illustrates the differences in PREDICT and NPI scores for patients in the ODX and non-ODX groups.

Impact of Oncotype DX testing on adjuvant chemotherapy treatment decisions

**Figure 3:**
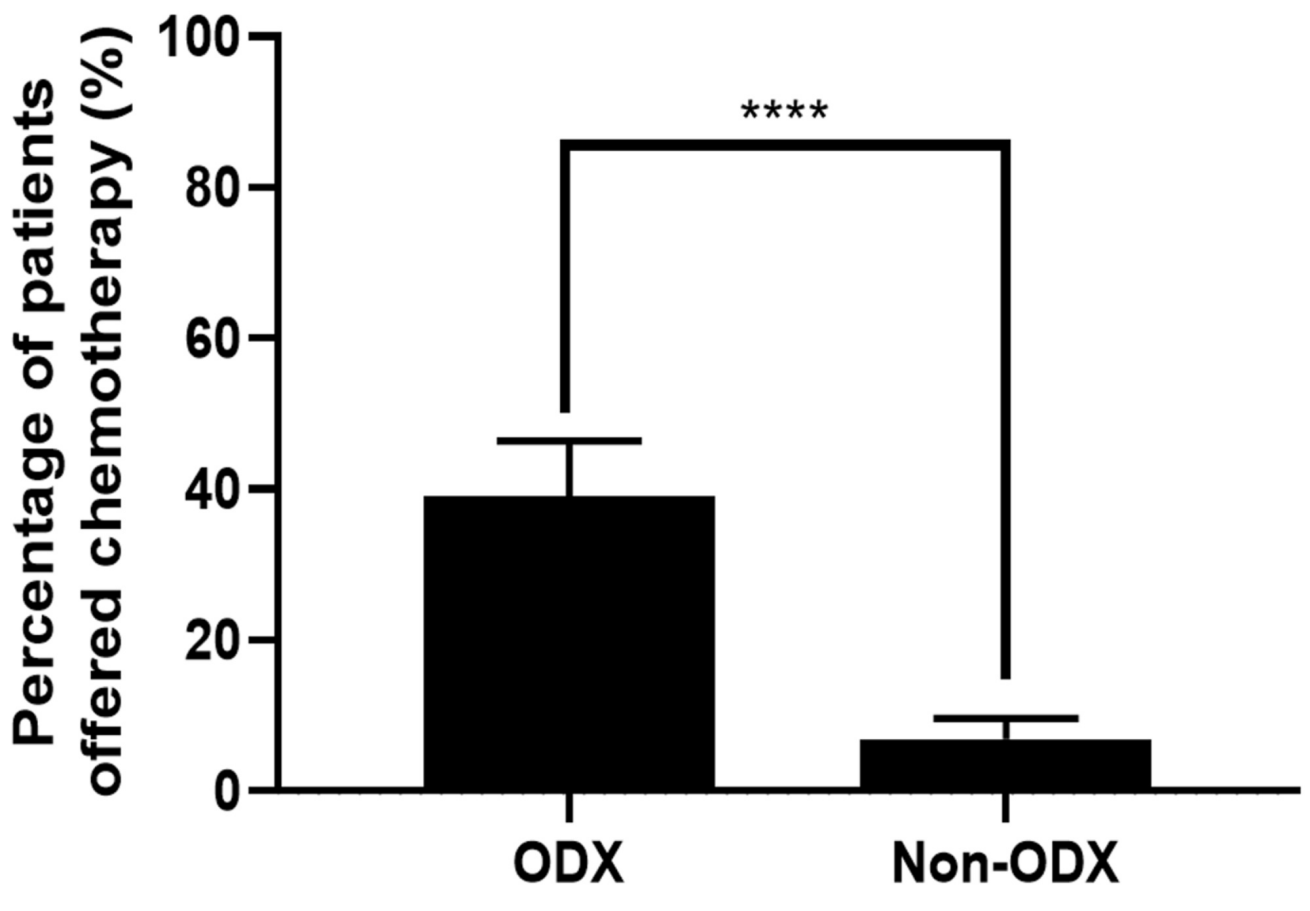
Chemotherapy treatment decisions for patients selected for Oncotype DX testing compared to patients not genetically profiled Statistical analysis: Mann-Whitney, P<0.0001; ODX group, patients with cancers that were genetically profiled through Oncotype DX testing; non-ODX group, patients that did not have Oncotype DX testing.

Based on the MDT decisions, 24/133 (18.0%) patients were offered adjuvant chemotherapy. Of these 24, 18 patients were offered chemotherapy after ODX testing, while six patients were offered chemotherapy without ODX testing. Figure 3 illustrates that a greater proportion of patients were offered chemotherapy in the ODX group in comparison to the non-ODXgroup (39.9% vs 6.9%, P<0.0001), independent of their NPI or PREDICT scores. In the ODX group, 3/46 (6.5%) patients with a low PREDICT score and 5/46 (10.9%) patients with a good NPI score were offered chemotherapy because they had a high RS (≥26). Of the 15 patients with high RS, chemotherapy was offered to all 15 patients; chemotherapy was also offered to 1/7 patients with an intermediate RS (18-25) and 2/24 patients with a low RS (<18). Low ODX scores resulted in the omission of chemotherapy in eight patients who would have otherwise been considered for adjuvant chemotherapy if the decision was based solely on an intermediate/high PREDICT score. 

Table 2 summarises the proportion of patients offered chemotherapy divided by PREDICT risk category. If all patients were considered, 30/133 (22.6%) had intermediate to high PREDICT risk (≥3) and could, therefore, have been considered candidates for chemotherapy or ODX testing based on their PREDICT score alone. Analysis of the patients offered chemotherapy in each PREDICT risk category revealed that patients with a low PREDICT score (<3) were significantly more likely to be offered chemotherapy after they underwent genetic profiling through ODX testing (22.7% vs 1.2%, P=0.0015). Conversely, in the intermediate PREDICT group, a lower proportion of patients were offered chemotherapy after ODX testing (54.5% vs 83.3%, P=0.3547), although this difference was not statistically significant.

**Table 2:** Adjuvant chemotherapy treatment decisions divided by PREDICT score for patients selected for Oncotype DX (ODX) testing compared to patients not genetically profiled *P values relate to Mann-Whitney tests comparing ODX and non-ODX groups; †, data excluded from statistical analysis due to non-positive values in non-ODX group. ODX group, patients with cancers that were genetically profiled through Oncotype DX testing; non-ODX group, patients that did not have Oncotype DX testing.

	Multidisciplinary team decision to offer chemotherapy			P-value*
	All patients	ODX Group	Non-ODX group	
All patients, N	24/133 (18.0%)	18/46 (39.1%)	6/87 (6.9%)	<0.001
Patients with low PREDICT (<3), n (%)	6/103 (5.8%)	5/22 (22.7%)	1/81 (1.2%)	0.0015
Patients with intermediate PREDICT (3-5), n (%)	17/28 (60.7%)	12/22 (54.5%)	5/6 (83.3%)	0.3547
Patients with high PREDICT (>5), n (%)	1/2 (50.0%)	1/2 (50.0%)†	0/0 (0.0%)†	

Table 3 summarises the proportion of patients offered chemotherapy in each group divided by NPI risk category. Of the 24 patients offered chemotherapy, significantly more patients with good NPI scores were likely to be offered chemotherapy after genetic profiling through ODX testing (28.6% vs 4.2%, P=0.0389). It is also worth noting that in the first NPI group, a higher proportion of patients were offered chemotherapy after ODX testing (16.7% vs 0.0%, P=0.1000), although this difference was not statistically significant. Overall, 28/133 (21.1%) patients had moderate to poor NPI risk. The majority of these 28 patients underwent ODX testing (n=19; 67.9%). However, the proportion of patients with a moderate NPI that were offered chemotherapy was similar between the ODX and non-ODX groups, with no statistical difference found (57.9% vs 55.6%, P>0.9999).

**Table 3:** Adjuvant chemotherapy treatment decisions divided by Nottingham Prognostic Index (NPI) score for patients selected for Oncotype DX (ODX) testing compared to patients not genetically profiled *P values relate to Mann-Whitney tests comparing ODX and non-ODX groups; †, data excluded from statistical analysis due to non-positive values in both groups; NPI, Nottingham Prognostic Index; ODX group, patients with cancers that were genetically profiled through Oncotype DX testing; non-ODX group, patients that did not have Oncotype DX testing.

	Multidisciplinary team decision to offer chemotherapy			P-value*
	All patients	ODX group	Non-ODX Group	
All patients, N	24/133	18/46	6/87	<0.0001
Patients with excellent NPI (≤2.4), n (%)	1/60 (45.1%)	1/6 (16.7%)	0/54 (0.0%)	0.1000
Patients with good NPI (2.41–3.4), n (%)	7/45 (33.8%)	6/21 (28.6%)	1/24 (4.2%)	0.0389
Patients with moderate NPI (3.41–5.4), n (%)	16/28 (21.1%)	11/19 (57.9%)	5/9 (55.6%)	>0.9999
Patients with poor NPI (≥5.41), n (%)	0/0 (0.0%)	0/0 (0.0%)†	0/0 (0.0%)†	

## Discussion

This study outlined our institution’s experience of using ODX testing to guide adjuvant chemotherapy treatment decisions for estrogen receptor-positive, HER2-negative, node-negative breast cancer. Our selection process reflects current national guidelines, with the majority of patients selected for ODX testing falling into the intermediate-risk PREDICT/NPI category, as recommended by NICE.

In the intermediate PREDICT group, a lower proportion of patients were offered chemotherapy after ODX testing. The final decision regarding the delivery of chemotherapy was based on the RS over a patient’s PREDICT or NPI score. This practice, which enabled the MDT to safely spare patients from adjuvant chemotherapy treatment, is a strategy supported by published data that suggests that ODX is superior to other clinicopathological factors at identifying patients who may not benefit from chemotherapy [12]. In addition, as a tool for risk stratifying women with ER-positive, node-negative breast cancer, ODX testing has been shown to be superior to the utilisation of clinicopathological factors [13-15].

However, some patients with low-risk PREDICT or good prognosis NPI scores were also selected from our cohort for ODX testing. A proportion of these patients had their risk category significantly increased after ODX testing and were consequently offered chemotherapy. A meta-analysis by Carlson et al. reported ODX testing changed treatment decisions for 49% of patients initially given low-risk recurrence scores by other methods [16]. This may be explained by observations that PREDICT underestimates breast cancer mortality in women with small ER-positive tumours and overestimates mortality in women with larger ER-positive tumours [17]. In addition, the presence of mitotically active tumour stroma and/or inflammatory cells associated with low-grade invasive breast carcinomas may contribute to higher RS scores generated by ODX testing in comparison to recurrence scores generated by clinicopathological scoring systems [18].

Indeed, the overall validity of clinicopathological scoring systems in heterogenous cohorts has been questioned [19-22]. A study by Plakhins et al. showed overall and breast cancer-specific survival obtained from PREDICT was significantly lower than the observed survival of the study population [19], while Quintyne et al. demonstrated that NPI also underestimates overall survival [19]. To account for this underestimation, studies have questioned the accuracy of reporting of clinicopathological factors, upon which the PREDICT and NPI scores are based. For example, Robbins et al. showed a statistically significant interobserver difference between pathologists in grading breast cancers. This study suggested that the concordance among pathologists in assessing tumour grades was ‘low’ for well-differentiated (low grade) tumours [23]. Therefore, it is not unreasonable practice for MDTs, at their discretion, to utilise ODX testing in patients who may appear low-risk based on their PREDICT/NPI, but who may have other concerning signs, symptoms or risk factors not considered by PREDICT or NPI.

There are clearly other important factors (such as comorbidities and patient choice) that play a role in deciding whether to offer adjuvant chemotherapy. It is, therefore, necessary to base decisions to select patients for ODX testing on more than only online prognostication tools, but rather to take a global view that incorporates recurrence risk scores along with clinicopathological factors, other histological features, fitness for chemotherapy, patient choice and performance status, when selecting patients for genetic profiling through ODX testing.

A potential limitation of our study was the fact that the patient population may have underrepresented ethnic minorities and young patients. Other potential limitations included the relatively small size of the cohort of patients that underwent ODX testing, which may impact on the generalisability of the results. To address this issue, future work should focus on assessing the impact of ODX testing in a larger cohort of patients across multiple centres.

## Conclusions

Patients selected for ODX testing at our institution were younger and had significantly higher-grade and larger-sized tumours than those that were not genetically profiled. The RS generated through ODX testing determined the final decision to offer adjuvant chemotherapy over clinicopathological tools such as PREDICT and NPI, thereby significantly impacting upon the adjuvant chemotherapy treatment decisions. Our results suggest that the MDT process consistently selects patients for ODX testing that presents with diagnostically uncertain or worrying features. Long-term recurrence and mortality data for our cohort would provide an invaluable basis for further evaluation of the selection process for genetic profiling.
